# lncRNA Xist Regulates Osteoblast Differentiation by Sponging miR-19a-3p in Aging-induced Osteoporosis

**DOI:** 10.14336/AD.2019.0724

**Published:** 2020-10-01

**Authors:** Shijie Chen, Yuezhan Li, Shuang Zhi, Zhiyu Ding, Yan Huang, Weiguo Wang, Ruping Zheng, Haiyang Yu, Jianlong Wang, Minghua Hu, Jinglei Miao, Jinsong Li

**Affiliations:** ^1^Department of Orthopaedics, The Third Xiangya Hospital of Central South University, Changsha, China.; ^2^Shanghai Key Laboratory of Regulatory Biology, Institute of Biomedical Sciences and School of Life Sciences, East China Normal University, Shanghai, China.; ^3^Department of Anatomy, Histology and Embryology, Changsha Medical University, Changsha, China.; ^4^Four Gynecological Wards, Ningbo Women & Children’s Hospital, Ningbo, Zhejiang, China.; ^5^The Second Xiangya Hospital of Central South University, Changsha, China.; ^6^School of Basic Medical Science, Central South University, Changsha, China.

**Keywords:** Osteoporosis, BMSCs, miR-19a-3p, Hoxa5, lncRNA Xist

## Abstract

The switch between osteogenic and adipogenic differentiation of bone marrow mesenchymal stem cells (BMSCs) plays a key role in aging-induced osteoporosis. In this study, miR-19a-3p was obviously downregulated in BMSCs from aged humans and mice. Overexpressed miR-19a-3p evidently reduced aging-induced bone loss in mice and promoted osteogenic differentiation of BMSCs, while silenced miR-19a-3p manifestly increased aging-induced bone loss in mice and repressed osteogenic differentiation of BMSCs. Hoxa5 was significantly downregulated in the BMSCs from aged mice and contribute to miR-19a-3p-induced osteoblast differentiation as a direct target gene of miR-19a-3p. Furthermore, lncRNA Xist was found as a sponge of miR-19a-3p to repress BMSCs osteogenic differentiation. In conclusion, our study reveals the critical role of the lncRNA Xist/miR-19a-3p/Hoxa5 pathway in aging-induced osteogenic differentiation of BMSCs, indicating the potential therapeutic target for osteoporosis.

Osteoporosis is a frequent age-related disease with severe bone loss, that affects millions of people in the world [1]. BMSCs could be differentiate into osteoblasts and adipocytes, and the switch showed more tendency to osteoblast with age, resulting in age-related marrow fat accumulation and progressive bone loss [2, 3]. It is, therefore, urgent for revealing the underline molecular mechanisms in aging-induced BMSCs differentiation.

MicroRNAs (miRNAs, with 18-24 nucleotides), a class of small noncoding RNAs that are reported to regulate multiple biological processes, including BMSCs differentiation. For example, Guo et al. revealed that the miR-23a/b was downregulated in aged BMSCs and contribute to osteogenic differentiation of BMSCs [4]. miR-204 was reported to be an attenuator of Runx2 and promote adipocyte differentiation of BMSCs [5]. miR-188 is evidently upregulated in aged BMSCs and induced age-related BMSCs adipocyte differentiation [3]. Despite these findings, the involvement of miRNA in BMSCs differentiation during aging-induced osteoporosis is still limited. miR-19a-3p (miR-19a) was reported as to be broadly conserved among vertebrates [6]. It was reported to participate in the pathogenesis of preeclampsia and atherosclerosis [7, 8]. A recent study has demonstrated the involvement of miR-19a-3p in the progression of various cancers including glioma, lung cancer, breast cancer, osteosarcoma, gastric cancer and hepatocellular carcinoma [9-13]. However, the precise mechanism by which miR-19a-3p in aging-induced osteoporosis remains unknown.

In this study, we identified that miR-19a-3p downregulated in the BMSCs of aged mice and humans and involves osteoporosis via promoting osteoblast differentiation from BMSCs by targeting Hoxa5. We also demonstrated that lncRNA Xist as the sponge of miR-19a-3p involved in osteoblast differentiation. In conclusion, our study clarified the Xist/miR-19a-3p/Hoxa5 in age-related BMSCs differentiation, and thus, might provide potential therapeutic approach for osteoporosis.

## MATERIALS AND METHODS

### Animals and Clinical samples

For miR-19a-3p^-/-^mice, we used CRISPR/Cas9 technology. In brief, we used the primer sequences of the miR-19a-3p F, 5′-CCCTGCTCCCTCTCTCAC-3′, and R, 5′-CAGAGAGCTCACCCTC-3′ for sgRNA plasmid. The mRNA was obtained from plasmid using the mMESSAGE (Life Technologies). The synthesized mRNAs were purified and injected to the zygotes from C57BL/6 mice as previous described [3]. For miR-19a-3p Tg mice, we synthesized pre-mir-19a-3p cDNA and cloned to an osterix vector to build an osterix-pre-miR-19a-3p plasmid. The plasmid was transfected into BMSCs and build the osteoprogenitor-specific miR-19a-3p transgenic mouse as previous described [3].

The bone marrow tissues were obtained from 61 female and 62 male patients with osteoarthritis undergoing knee joint replacement or with femoral neck fracture and/or femoral head fractures undergoing hip joint replacement, (with age from 20 to 85) from the Department of Orthopaedics of the Third Xiangya Hospital. The present study was approved by the e Animal Care and Use Committee and Ethics Committee of the Third Xiangya Hospital of Central South University.

### BMSC Culture and Transfection

The BMSCs were isolated from mouse and human as previous described [4, 14]. The agomiR-19a-3p and antagomiR-19a-3p, the plasmid and siRNA of Hoxa5 and Xist were synthesized from Genepharma (Suzhou, China). agomiRNA, antagomiRNA, plamis and siRNA were transfected to BMSCs as previous described [4].

**Table 1:** The primers.

	Forward 5’-3’	Reverse 5’-3’
*β-actin*	CTGTCCCTGTATGCCTCTG	TGATGTCACGCACGATTT
*Runx2*	ACTTCCTGTGCTCCGTGCTG	TCGTTGAACCTGGCTACTTGG
*Hoxa5*	CTCATTTTGCGGTCGCTATCC	ATCCATGCCATTGTAGCCGTA
*Osterix*	ACCAGGTCCAGGCAACAC	GCAAAGTCAGATGGGTAAGTAG
miR-19a-3p	CTGGAGTGTGCAAATCTATGC	GTGCAGGGTCCGAGGT
LncRNA Xist	AATGGAACGGGCTGAGTTTTAG	TCATCCGCTTGCGTTCATAG
U6	GCGCGTCGTGAAGCGTTC	GTGCAGGGTCCGAGGT
Antagomir-19a-3p	TCAGTTTTGCATAGATTTGCACA	
antagomir NC	CAGUACUUUUGUGUAGUACAA	
agomir-19a-3p	UGUGCAAAUCUAUGCAAAACUGA	
pGL3-HOXA5	GGGGTACC GCATCTGAGCG	CCGCTCGAG GCTGATCACAGTT
pGL3-Xist	GGGGTACCCGGCTTGCTCCA	CCGCTCGAG TTCAAAACAAAGCA
pcDNA3.1-HOXA5	GGGGTACC ATGAGCTCTTATT	CCGCTCGAG TCAGGGGCGGAA
pcDNA3.1-Xist	GGGGTACCCGGCTTGCTCCA	CCGCTCGAG TTCAAAACAAAGCA
siHOXA5	GCACATTAGTCACGACAAT	
si_control	GCATTAGTCACGACCAAAT	
siXist	GCACACATCTCATTCCATT	

### Microcomputed Tomography Analysis

The right femora from young and old mice were scanned and analyzed by high-resolution mCT (Skyscan 1172, Skyscan). For the distal femur, we selected the region of interest (ROI) for detecting the trabecular number (Tb. N), trabecular thickness (Tb. Th), trabecular separation (Tb. Sp), trabecular bone volume per tissue volume (Tb. BV/TV). To examine dynamic bone formation, calcein double labelling was used for trabecular bone, bone formation rates (BFRs) and mineral apposition rate (MAR) as previous described [14].

### Osteogenic Differentiation

5×10^5^ BMSCs were seeded into 24-well plates to induce osteogenic differentiation using osteogenic-inducing medium as previous described. We used the alkaline phosphatase (ALP) kit (Roche Diagnostics, Minneapolis, MN, USA) for ALP activity assay and used the specific immunoassay kit (DiaSorin, Stillwater, MN, USA) for detecting osteocalcin levels. Alizarin Red staining was used to detected osteoblastic mineralization of BMSCs as previous described [14].

### qRT-PCR Analysis

qRT-PCR was used for detecting gene, miRNA or lncRNA expression as previously described [15]. The nucleotide sequences of primers for miR-19a-3p, and U6, Runx2, Osterix, ITGB3 and β-actin are listed in Table 1.

### Western Blot

Total protein was obtained from BMSCs and then separated using SDS-PAGE, and then incubated anti-Hoxa5 antibody (sigma, USA) on PVDF membranes (Millipore) 4 ? overnight followed by HRP conjugated secondary antibody for 1 hour at room temperature. The protein expression levels were analyzed using using ChemiDoc XRS + Imaging System (BioRad).

### Dual Luciferase Reporter Assay

We cloned the 3′- UTR of Hoxa5 into pGL3 luciferase reporter vector (Promega, Madison, WI, USA). And the mut-HOXA5 pGL3 was obtained by site-directed mutagenesis method. The HOXA5-pGL3 or mutHOXA5-pGL3 was contransfected with agomiR-19a-3p or agomiR-NC, or antagomiR-19a-3p or antagomiR-NC into BMSCs and the Dual-Luciferase Reporter Assay System (Promega) was used to detecte the luciferase activities.

For the relationship between Xist and miR-19a-3p, we cloned the potential binding site between miR-19a-3p and Xist to pGL3 vector, and then build a mutant XIST-pGL3 using site-directed mutagenesis method. The XIST-pGL3 or mutXIST-pGL3 was contransfected with agomiR-19a-3p or agomiR-NC, or antagomiR-19a-3p or antagomiR-NC into BMSCs and the luciferase activities was detected using Dual-Luciferase Reporter Assay System (Promega).

### RNA Immunoprecipitation (RIP) Assay

pMS2bp-GFP and MS2, MS2-XIST, or MS2- mutXIST were contransfected to BMSCs cells, and harvested at 48 hours after transfection. The biotin-coupled RNA complex was pulled down and was used for RIP assay using Magna RIP™ Kit (Millipore, USA), according to the manufacturer’s instructions [16]. The anti-GFP and anti-IgG were used to incubate, and qPCR was used for the miR-19a-3p level.

**Figure 1.**
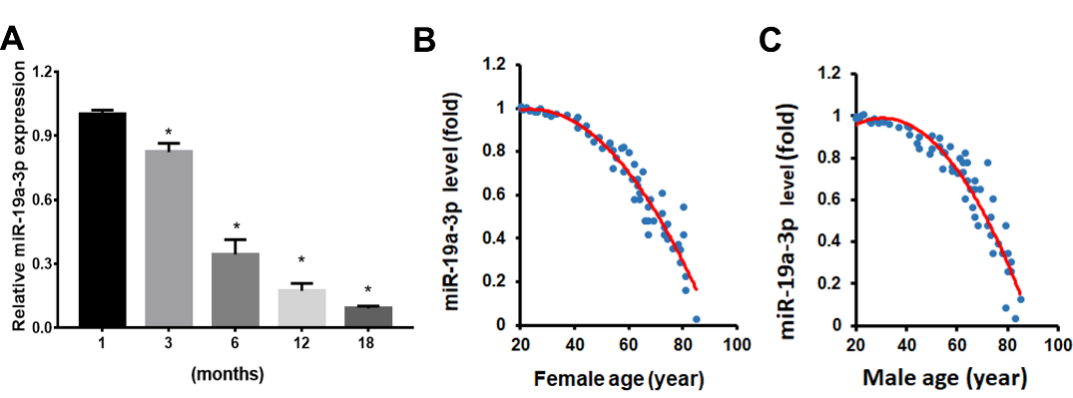
Aging induces miR-19a-3p expression in BMSCs. A The miR-19a-3p expression in BMSCs from mice with different ages. B, The relationship between miR-19a-3p expression with age in BMSCs from 61 human females and C, 62 males. Data shown as mean ± SD. *p < 0.05.

### RNA Pull-down Assay

The capture of miR-19a-3p- bound lncRNAs in a pull-down assays with biotinylated miR-19a-3p and biotinylated lncRNA Xist were performed as previously described[16]. The Biotin-labeled miR-19a-3p and Xist were transfected into BMSCs. After 72 h, BMSCs were harvested and the biotin-coupled RNA complex was pulled down by incubating the cell lysates with M-280 streptavidin magnetic beads (Sigma) for binding at 4 ? for at least 3 h. And then, the beads bounded with RNA was purified by TRIzol. qPCR assay was used for detecting Xist or miR-19a-3p levels.

### FISH

Briefly, FITC-labeled Xist and Cy3-labeled miR-19a-3p were designed and synthesized by Songan Biotech. The probe signals were detected using a situ hybridization kit (RiboBio) for FISH assay according to the manufacturer’s instructions as previous described [17]. The Leica SP5 confocal microscope (Leica Microsystems) was used for confocal images.

### Statistical Analyses

All data were analyzed using SPSS 22.0 and presented as the mean ± s.d. A Student’s *t*-test was used for the comparisons of two groups. One-way ANOVA was used for the comparisons of multiple groups. *P*<0.05 presents statistically significant.

## RESULTS

### miR-19a-3p Expression in BMSCs with Age

Previous studies showed that BMSCs observed more tendency to adipocytes differentiate with age. To investigate the dysregulated miRNA expression in aged BMSCs, we analyzed the miRNA expression from GEO database (accession number GSE57127) and found that miR-19a-3p were downregulated in aged BMSCs. We next verified the miR-19a-3p expression in BMSCs from the mice with different ages using qPCR assay (Fig. 1A). We confirmed the decreased miR-19a-3p levels in human BMSCs with aging and revealed the negative relationship between miR-19a-3p expression and age (Fig. 1B and C). This result revealed the potential important role of miR-19a-3p in the aging process BMSCs.

**Figure 2.**
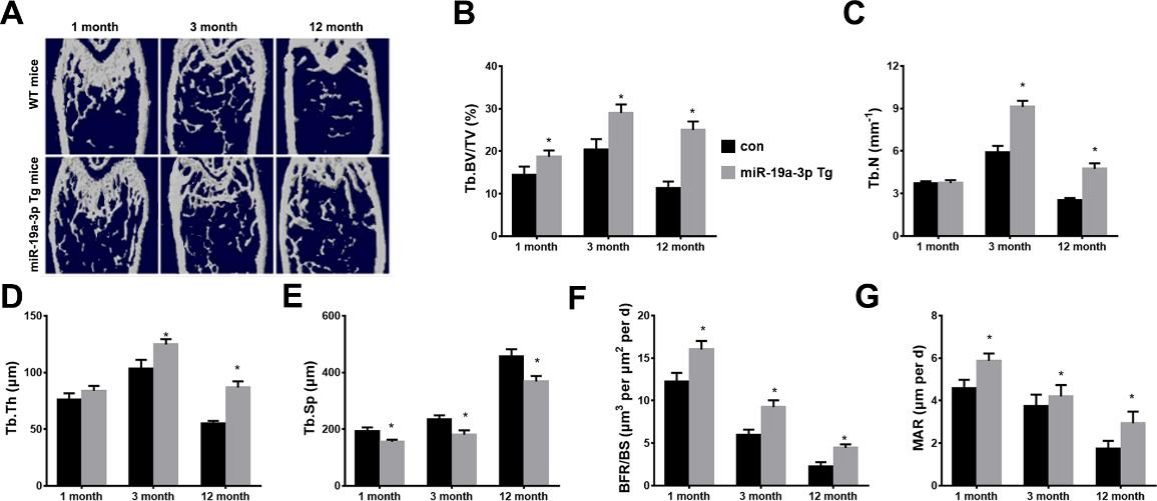
The age-associated bone loss in miR-19a-3p Tg mice. (A) The μCT images of WT mice and miR-19a-3p Tg mice at 1, 3 and 12 months old. B-E, quantitative μCT analysis of trabecular bone microarchitecture. (B) Tb. BV/TV, trabecular bone volume per tissue volume. (C) Tb.N, trabecular number. (D) Tb.Th, trabecular thickness. E, Tb.Sp, trabecular separation. (F, G) Representative images of calcein double labelling of trabecular bone, (G) with quantiﬁcation of BFR per bone surface BFR/BS, H, and mineral apposition rate MAR. Data shown as mean ± SD, *p <0.05.

### Overexpressed miR-19a-3p attenuates age-associated bone loss

To explore the involvement of miR-19a-3p in age-related osteoporosis, the 1, 3, 12 months old WT mice and transgenic overexpressing miR-19a-3p (miR-19a-3p Tg) mice was used to detected aged-associated bone loss using Microcomputed tomography (μ-CT). We found that the trabecular bone volume, number and thickness were significantly increased, and the trabecular separation was evidently decreased in miR-19a-3p Tg mice of aged (3 or12 months) compared to WT controls (Fig. 2A-E). Moreover, miR-19a-3p Tg mice showed increased bone formation rates (BFRs; Fig. 2F and G). These results suggested that highly expressed miR-19a-3p attenuates the aging-induced bone loss.

### Knockout miR-19a-3p Induces Age-associated Bone Loss

**Figure 3.**
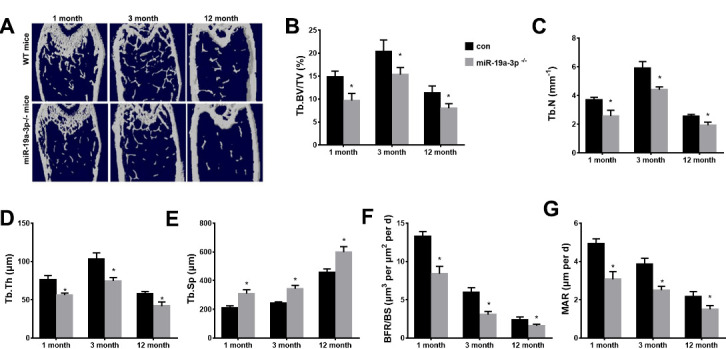
Age-associated bone loss in miR-19a-3p knockout mice. (A) μCT images of WT mice and miR-19a-3p^-/-^mice at 1, 3 and 12 months old. B-E, quantitative the trabecular bone microarchitecture. (B) Tb. BV/TV, trabecular bone volume per tissue volume. (C) Tb.N, trabecular number. (D) Tb.Th, trabecular thickness. (E) Tb.Sp, trabecular separation. (F, G) quantification of BFR/BS and MAR. (F) with quantiﬁcation of BFR per bone surface (BFR/BS); (G) mineral apposition rate (MAR). Data shown as mean ± SD. *p <0.05.

We next used knockout mice (miR-19a-3p^-/-^mice) to investigate whether silenced miR-19a-3p induces age-associated bone loss in vivo. As shown in Figure 3A-E, the trabecular bone volume, the trabecular bone number and the trabecular bone thickness were significantly decreased in the femora of 3-month-old and 12-month-old miR-19a-3p^-/-^mice relative to WT controls. The trabecular separation was evidently increased in miR-19a-3p^-/-^mice of aged (3 or 12 months) compared to WT controls. Moreover, bone formation rates were also reduced in miR-19a-3p^-/-^mice as compared with WT controls (Fig. 3F and G). Together, our results suggested that silenced miR-19a-3p promoted the aging-induced bone l.

### miR-19a-3p Regulates the BMSCs Osteogenic Differentiation

**Figure 4.**
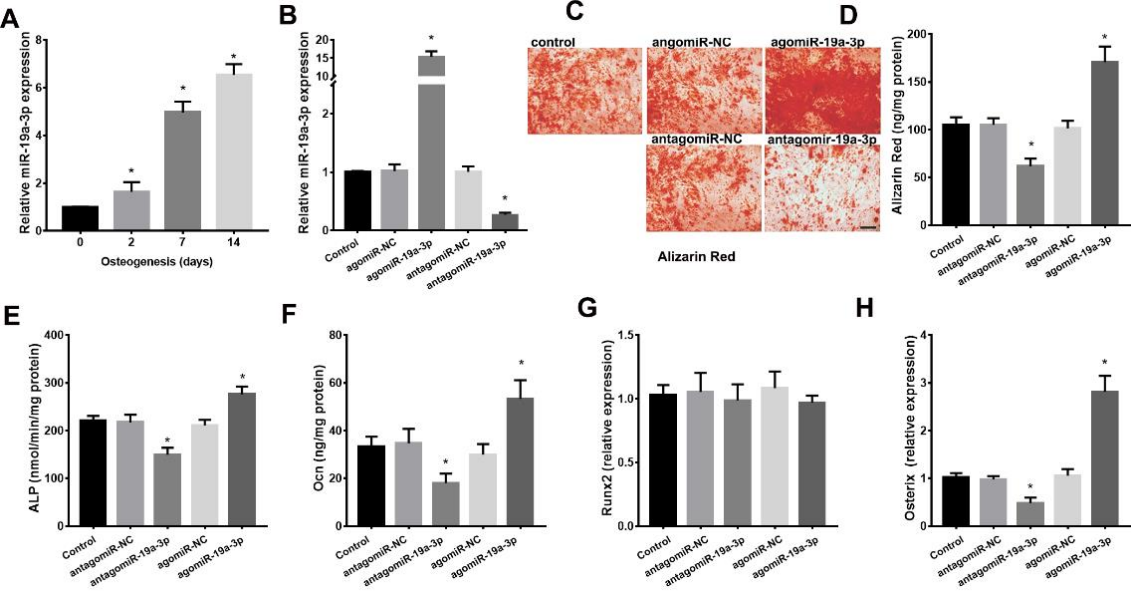
miR-19a-3p regulates the osteogenic differentiation in BMSCs. (A) miR-19a-3p expression in the BMSCs during osteogenic differentiation. (B) The antagomiR-19a-3p or agomiR-19a-3p were used to silence or overexpress miR-19a-3p in BMSCs. (C, D) Alizarin Red staining revealed the role of miR-19a-3p on the osteogenic differentiation of BMSCs. Scale bars: 100 μm. (E) ALP activity, (F) osteocalcin secretion, (G) RUNX2 and H, osterix expression in BMSCs. Data shown as mean ± SD. *p <0.05.

We next detected role of miR-19a-3p on osteogenic differentiation of BMSCs. As shown in Figure 4A, miR-19a-3p expression was gradually increased during osteogenic differentiation in the BMSCs from 8 weeks-old mice. We next silenced or overexpressed miR-19a-3p in BMSCs using antagomiR-19a-3p or agomiR-19a-3p (Fig. 4B). Alizarin Red staining demonstrated the reduction of osteogenic differentiation in miR-19a-3p silenced BMSCs, whereas the increase of osteogenic differentiation in the miR-19a-3p overexpressed BMSCs (Fig. 4C and D). Consistently, the markers of osteoblast differentiation, including ALP activity, osteocalcin secretion, osteoblast transcription factor osterix were increased by overexpressed-miR-19a-3p but decreased by silenced-miR-19a-3p (Fig. 4E-H). However, over-expressed-miR-19a-3p or silenced-miR-19a-3p did not significant affect the expression of RUNX2 in BMSCs (Fig. 4G). These results indicated that miR-19a-3p promotes the osteogenic differentiation in BMSCs.

### miR-19a-3p Directly Targets Hoxa5

**Figure 5.**
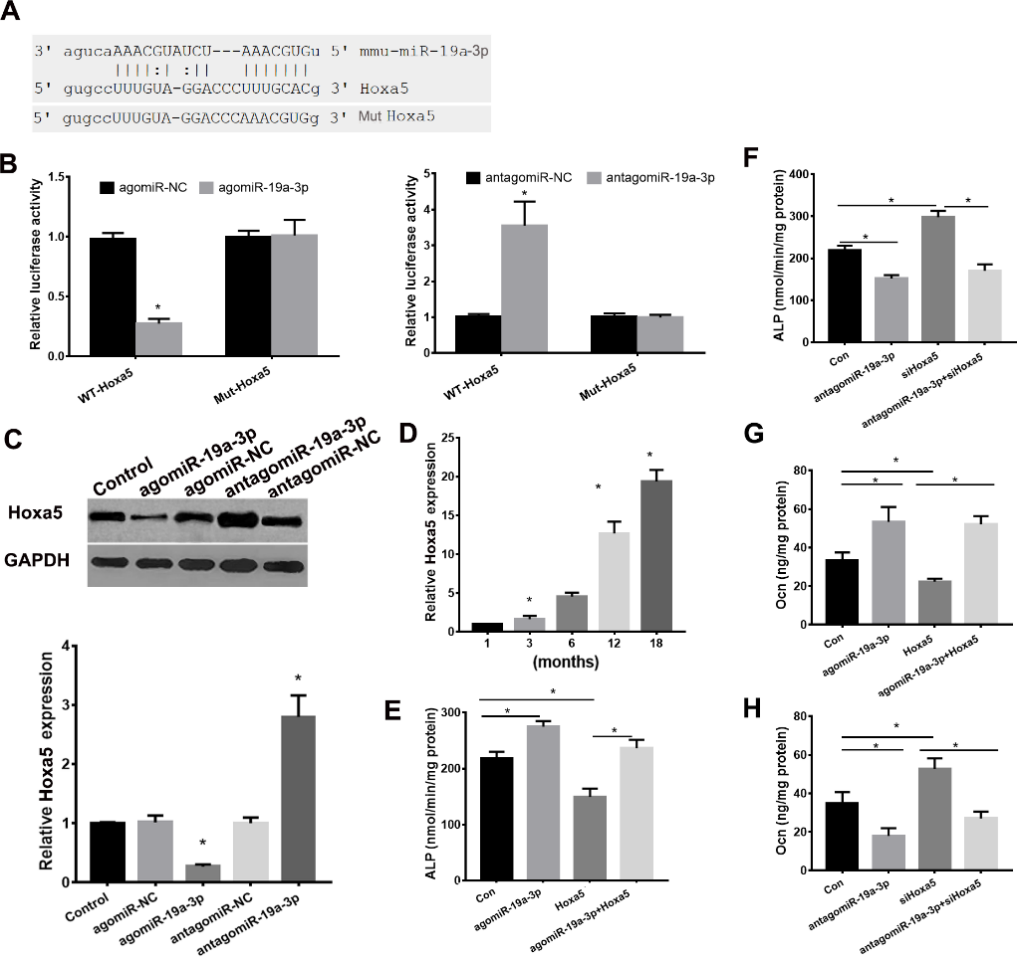
miR-19a-3p regulated Hoxa5 expression by targeting the 3’UTR of Hoxa5 directly. (A) the predict binding role of miR-19a-3p on 3′-UTR of Hoxa5. (B) luciferase reporter assay revealed the target role of miR-19a-3p on Hoxa5. (C) miR-19a-3p regulated Hoxa5 expression. (D) Hoxa5 expression was increased in BMSCs of the aging mice. (E, F) The role of Hoxa5 and miR-19a-3p on ALP activity of BMSCs. (G, F) The role of Hoxa5 and miR-19a-3p on osteocalcin secretion of BMSCs. Data shown as mean ± SD. *p <0.05.

miRNAs regulate mRNAs expression by binding to the 3′-UTRs of mRNA [13]. We next used microrna (www.microrna.org/microrna/home.do) and targetscan (/www.targetscan.org) to predict the target genes of miR-19a-3p. We found that homeobox protein A5 (Hoxa5), a regulator of osteogenic differentiation, was predicted target of miR-19a-3p (Fig. 5A), so we choose Hoxa5 for the further analysis. Next, we used a luciferase reporter assay to clarify the role of miR-19a-3p on Hoxa5. As shown in Fig. 5B, agomiR-19a-3p evidently reduced the luciferase activity of WT-pGL3-HOXA5 but fail to affect the luciferase activity of MUT-pGL3-HOXA5 in BMSCs. antagomiR-19a-3p increased the luciferase activity of WT-pGL3-HOXA5 but failed to affect the luciferase activity of MUT-pGL3-HOXA5 in BMSCs. These results confirmed that miR-19a-3p targets Hoxa5 at its*3′*-UTR directly. We further determined the role of miR-19a-3p on Hoxa5 expression. We found that silenced miR-19a-3p evidently elevated both mRNA and protein expression levels of Hoxa5, whereas overexpressed miR-19a-3p repressed the mRNA and protein levels of Hoxa5 (Fig. 5C). Moreover, Hoxa5 expression was increased in BMSCs of aged mice (Fig. 5D). The Hoxa5 expression was downregulated in the BMSCs from aged (12 months) miR-19a-3p tg mice and upregulated in the BMSCs from aged (12 months) KO mice (Supplementary Fig. 1A).

We next determine the role of Hoxa5 on miR-19a-3p-mediated repression of osteogenic differentiation in BMSCs. We found that overexpressed Hoxa5 reduced ALP activity and osteocalcin secretion (Fig. 5E and G), overexpressed Hoxa5 reduced agomiR-19a-3p-induced ALP activity and osteocalcin secretion in BMSCs. Moreover, silenced Hoxa5 markedly induced ALP activity and silenced Hoxa5 reversed antagomiR-19a-3p-mediated repression of ALP activity and osteocalcin secretion (Fig. 5F and H). In conclusion, these data indicated that miR-19a-3p regulates age-associated BMSC differentiation by targeting Hoxa5 expression directly.

### lncRNA Xist Acted as a Sponge of miR-19a-3p

**Figure 6.**
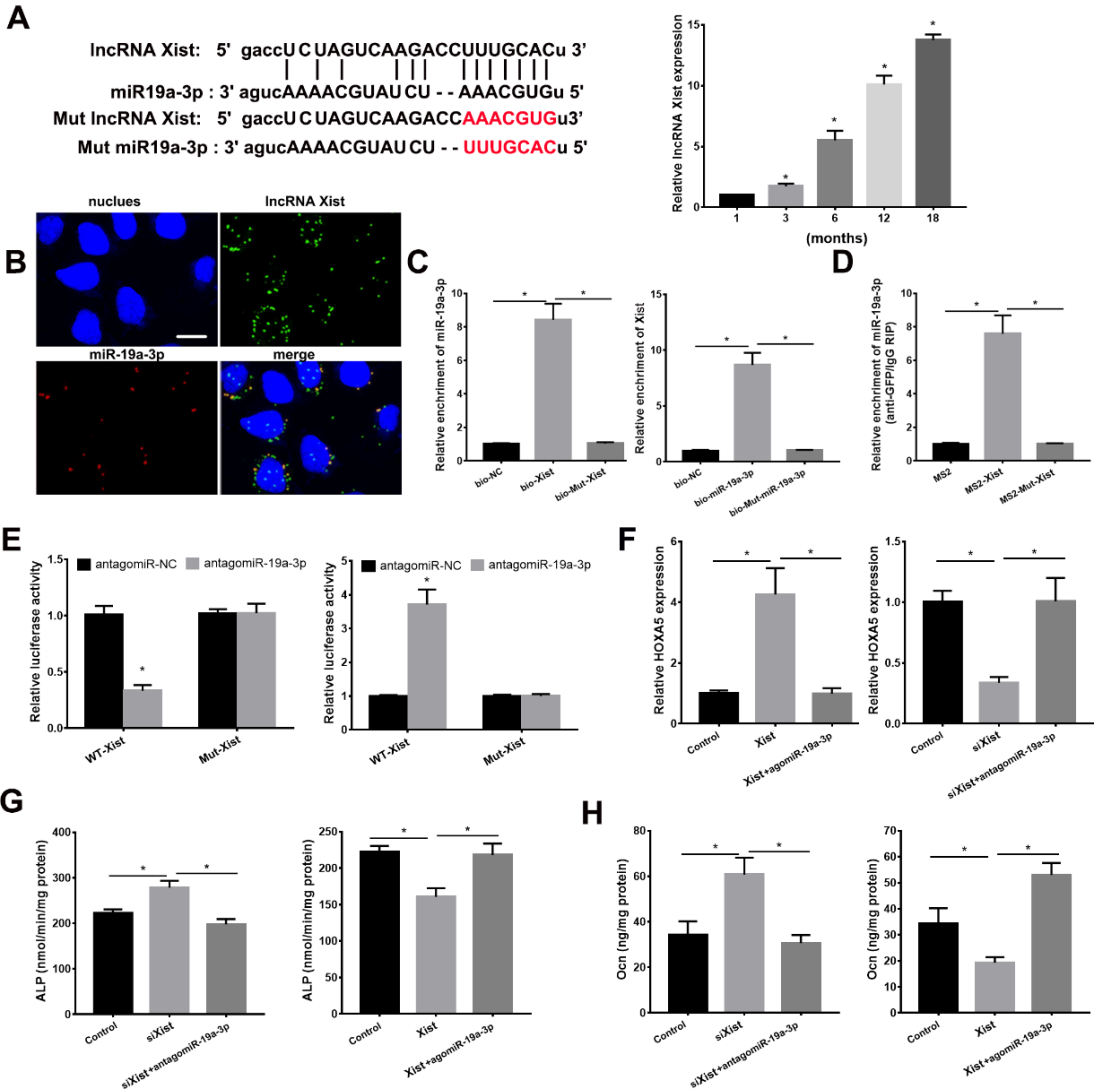
LncRNA Xist was involved osteoblast differentiation via miR-19a-3p/Hoxa5 pathway. (A) Xist expression in BMSCs from mice with different age. (B) RNA FISH assay for miR-19a-3p and Xist. Scale bar=10 µm. (C) BMSCs were incubated with biotin-labeled miR-19a-3p and Xist, and qPCR analyses revealed the production of pulled down. (D) RIP assay to reveal the relationship between miR-19a-3p and Xist. (E) Luciferase reporter assay revealed the target role of miR-19a-3p and Xist. (F) The role of Xist and miR-19a-3p on Hoxa5 expression in BMSCs. (G) The effects of Xist and miR-19a-3p on ALP activity. H, The role of Xist and miR-19a-3p on osteocalcin secretion in BMSCs. Data shown as mean ± SD. *p <0.05.

We next predict the lncRNA which may bind to miR-19a-3p using starBase. As shown in Figure 6A, miR-19a-3p may be targeted by lncRNA Xist. The lncRNA Xist expression in BMSCs with different age was detected using qPCR analysis. As the animals aged, lncRNA Xist expression was increased in BMSCs (Fig. 6A). RNA FISH revealed the colocalization of lncRNA Xist and miR-19a-3p in the cytoplasm of BMSCs in Figure 6B. We performed RNA pull-down assay and RNA immune-precipitation (RIP) assay to further verify the relationship between miR-19a-3p and Xist. As shown in Figure 6C, miR-19a-3p was pulled down by biotin-labeled WT XIST but failed to be pulled down by mut-XIST. Xist was pulled down by biotin-labeled WT miR-19a-3p, which was failed by mutation miR-19a-3p (Fig. 6C). The RIP assay showed that miR-19a-3p was enriched in Xist, but the enrichment was not affected in mut Xist group. These data showed that miR-19a-5p targets Xist dierctly. We next used the luciferase assay to confirm the target role between lncRNA Xist and miR-19a-3p. As shown in Figure 6E, miR-19a-3p evidently reduced the luciferase activities in Xist group but failed to affect the luciferase activity in mut Xist group. The Xist expression was similar in BMSCs from aged (12 months) WT, miR-19a-3p tg and KO mice (Supplementary Fig. 1B). Together, these results indicated the direct binding role between lncRNA and miR-19a-3p in BMSCs.

To determine the regulation of lncRNA Xist on Hoxa5, we transfected pcDNA3.1-XIST to overexpress Xist in miR-19a-3p-overexpressed BMSCs and transfected siRNA Xist to silence Xist in miR-19a-3p-knockdown BMSCs. As shown in Figure 6F, overexpressed Xist evidently induced Hoxa5 expression, which was reduced by overexpressed miR-19a-3p. Conversely, silenced Xist reduced Hoxa5 expression, which was abrogated by silenced miR-19a-3p. Taken together, these above results indicated that lncRNA Xist acted as a functional sponge of miR-19a-3p to regulate Hoxa5 expression in BMSCs. Next, we investigated the function of lncRNA Xist in osteoblast differentiation, by detecting ALP activity and osteocalcin secretion. Enforced Xist reduces ALP activity and osteocalcin secretion of BMSCs, which was abolished by overexpressed miR-19a-3p (Fig. 6G and H). Conversely, silenced Xist induced ALP activity and osteocalcin secretion, which was reversed by antagomiR-19a-3p (Fig. 6G and H). These data collectively indicated that lncRNA Xist was participated in osteoblast differentiation via miR-19a-3p/Hoxa5 pathway.

## DISCUSSION

BMSCs plays an important role on bone homeostasis via differentiating into osteoblasts or adipocytes. However, the BMSCs tend to differentiate into adipocytes with age, which results in age-related osteoporosis with increased fat and bone loss. Here, we described that miR-19a-3p involved in aging-induced osteoporosis via regulating Hoxa5-mediated BMSCs differentiation.

Recently, several miRNAs have been implicated in the pathogenesis of aged-related osteoporosis [18, 19]. However, the mechanism of miRNAs in aging-induced BMSCs differentiation during osteoporosis remains limited. MiR-19a-3p is a members of the miR-17-92 cluster, and usually reported as oncogene in various cancers, including lung cancer [13, 20], colorectal cancer [21], breast cancer [10], hepatocellular carcinoma[12] and cervical cancer [22]. Studies also showed that miR-19a-3p involved in the vascular inflammation of atherosclerosis [7] and involved in the epithelium repair of asthma by regulating proliferation of bronchial epithelial cells [23]. Recently, study demonstrated miR-19b-3p as an important predictive marker of circulating miRNA for aging [24]. miR-17, a member of miR-17-92 cluster, was reported to act as positive regulator of osteogenesis in an inflammatory microenvironment [25]. However, the potential role of miR-19a-3p in aging-induced osteoporosis still remain elusive. In this study, miR-19a-3p expression was remarkably reduced in BMSCs from aged mice and human participants. Furthermore, miR-19a-3p^-/-^ mice worsen aging-induced osteoporosis with increased bone loss, and miR-19a-3p transgenic mice attenuated aging-induced bone loss. Moreover, miR-19a-3p induced the osteogenic differentiation of BMSCs in vitro. These results demonstrated the important role of miR-19a-3p in aging-induced osteoporosis by regulating BMSCs osteogenic differentiation.

miRNA was reported to involve in various biological processes through regulating the expression of mRNA by targeting their 3’ UTR. Previously, miR-19a-3p was involved in tumorigenesis of cervical carcinoma by targeting CUL5 [22]. miR-19a-3p also reported to be involved in the proliferation of colorectal cancer by targeting TIA1 [26] and regulates progression of glioma by targeting RUNX3 directly [27]. Recent study showed that miR-19a-3p regulated macrophage polarization by targeting Fra-1 [6]. Here, we revealed that Hoxa5 was direct target of miR-19a-3p and contribute to miR-19a-3p-induced BMSC osteogenic differentiation. Hoxa5 was served as tumor suppressor in a number of cancers including non-small cell lung cancer [28], osteosarcoma [29], gastric cancer [30] and gastric cancer [31]. Hoxa5 was also reported to alleviate inflammation and induce adipose tissue browning [32]. Recently, it was shown that Hoxa5 promoted adipocytes differentiation in primary adipocytes [33]. In this study, we reported that Hoxa5 participated in the miR-19a-3p-mediated switch of BMSCs to osteoblasts.

lncRNAs, a class of >200 nucleotides non-coding RNAs, was reported to be involved in various progression of disease including osteoporosis [34-36]. For example, Yang et al. showed that lncRNA ORLNC1 involved in osteoporosis via regulating BMSCs osteogenic and adipogenic differentiation [37]. Liu et al. showed that Lnc-AK077216 promotes osteoclastogenesis via regulating NFATc1 expression [36]. Wang et al. demonstrated lncRNA ODSM as a regulator of osteoblast differentiation and apoptosis via targeting miR-139-3p in osteoblasts [19]. Xist was initially reported as a lncRNA, involving in X chromosome inactivation [38]. Increasing evidences showed that lncRNA Xist was reported as an oncogene in various tumors [39, 40]. Moreover, recent studies indicated the involvement of lncRNA Xist in myocardial infarction [41]. In this study, lncRNA Xist serve as a sponge of miR-19a-3p in BMSCs, and lncRNA Xist regulated Hoxa5 expression and BMSCs osteogenic differentiation via targeting miR-19a-3p directly.

In conclusion, our finding indicated that miR-19a-3p expression was downregulated in BMSCs with aged and the exogenous miR-19a-3p in BMSCs promoted osteogenic differentiation and bone formation partly by targeting Hoxa5 directly. Moreover, lncRNA Xist function as the sponge of miR-19a-3p and was involved in osteogenic differentiation. These results demonstrated the function of the lncRNA Xist/miR-19a-3p /Hoxa5 signaling pathway in aging-induced BMSCs osteogenic differentiation, revealing a potential therapeutic target for osteoporosis.

## Supplementary Materials

The Supplemenantry data can be found online at: www.aginganddisease.org/EN/10.14336/AD.2019.0724.
