# Supplementary material for: lncRNA Xist Regulates Osteoblast Differentiation by Sponging miR-19a-3p in Aging-induced Osteoporosis
Source: Aging Dis. 2020 Oct 1;11(5):1058–68. doi: 10.14336/AD.2019.0724 (PMC7505278; doi:10.14336/AD.2019.0724)
Supplement: Supplementary file 1 — The Supplemenantry data can be found online at: www.aginganddisease.org/EN/10.14336/AD.2019.0724. [file AD-11-5-1058-suppl.pdf]

SUPPLEMENTARY DATA

lncRNA Xist Regulates Osteoblast Differentiation by Sponging miR-19a-3p in Aging-induced Osteoporosis

Shijie Chen[1, 2], Yuezhan Li[1], Shuang Zhi[4], Zhiyu Ding[1], Yan Huang[5], Weiguo Wang[1], Ruping Zheng[6], Haiyang Yu[6], Jianlong Wang[1], Minghua Hu[3], Jinglei Miao[1*], Jinsong Li[1*]

[1]Department of Orthopaedics, The Third Xiangya Hospital of Central South University, Changsha, China. [2]Shanghai Key Laboratory of Regulatory Biology, Institute of Biomedical Sciences and School of Life Sciences, East China Normal University, Shanghai, China. [3]Department of Anatomy, Histology and Embryology, Changsha Medical University, Changsha, China. [4]Four Gynecological Wards, Ningbo Women & Children's Hospital, Ningbo, Zhejiang, China. [5]The Second Xiangya Hospital of Central South University, Changsha, China. [6]School of Basic Medical Science, Central South University, Changsha, China.

© 2019. Chen S et al. Published online at http://www.aginganddisease.org/EN/10.14336/AD.2019.0724

SUPPLEMENTARY DATA

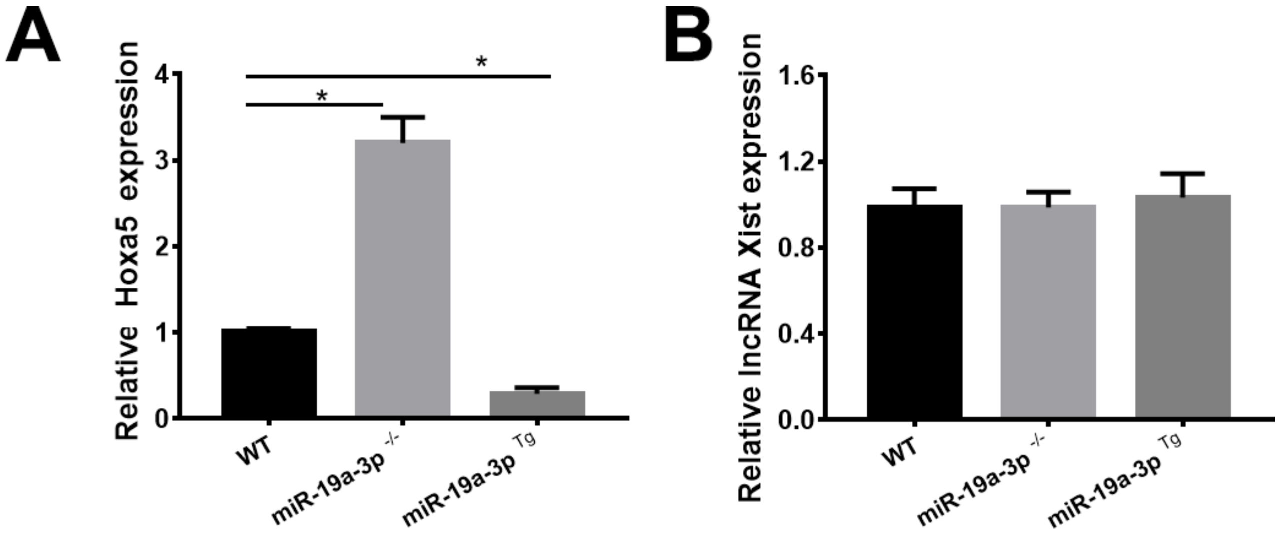

Supplementary Figure 1. The expression of Hoxa5 and Xist in BMSCs from the miR-19a-3p tg and KO mice. (A) The mRNA expression of Hoxa5 in BMSCs from the miR-19a-3p tg and KO mice. **(B)** The expression of Xist in BMSCs from the miR-19a-3p tg and KO mice. Data shown as mean ± SD. *p <0.05.

© 2019. Chen S et al. Published online at http://www.aginganddisease.org/EN/10.14336/AD.2019.0724
